# Trends in Heart-Rate Variability Signal Analysis

**DOI:** 10.3389/fdgth.2021.639444

**Published:** 2021-02-25

**Authors:** Syem Ishaque, Naimul Khan, Sri Krishnan

**Affiliations:** Department of Electrical, Computer and Biomedical Engineering, Ryerson University, Toronto, ON, Canada

**Keywords:** heart rate variability, wireless sensors, drowsiness, stress, morbidity, exercise, machine learning

## Abstract

Heart rate variability (HRV) is the rate of variability between each heartbeat with respect to time. It is used to analyse the Autonomic Nervous System (ANS), a control system used to modulate the body's unconscious action such as cardiac function, respiration, digestion, blood pressure, urination, and dilation/constriction of the pupil. This review article presents a summary and analysis of various research works that analyzed HRV associated with morbidity, pain, drowsiness, stress and exercise through signal processing and machine learning methods. The points of emphasis with regards to HRV research as well as the gaps associated with processes which can be improved to enhance the quality of the research have been discussed meticulously. Restricting the physiological signals to Electrocardiogram (ECG), Electrodermal activity (EDA), photoplethysmography (PPG), and respiration (RESP) analysis resulted in 25 articles which examined the cause and effect of increased/reduced HRV. Reduced HRV was generally associated with increased morbidity and stress. High HRV normally indicated good health, and in some instances, it could signify clinical events of interest such as drowsiness. Effective analysis of HRV during ambulatory and motion situations such as exercise, video gaming, and driving could have a significant impact toward improving social well-being. Detection of HRV in motion is far from perfect, situations involving exercise or driving reported accuracy as high as 85% and as low as 59%. HRV detection in motion can be improved further by harnessing the advancements in machine learning techniques.

## 1. Introduction

HRV has been associated with many research studies involving morbidity and mortality, stress, fatigue and athletic performance. HRV is primarily used to assess the function of the autonomic nervous system (ANS), it consists of the sympathetic nervous system (SNS) and the parasympathetic nervous system (PNS) which coordinates the activities of the body's unconscious actions as a part of the peripheral nervous system. SNS is known as the fight and flight response, it operates within the middle of the spinal cord and activates in response to stress causing an increase in HR, constriction of blood vessels and an increase in blood pressure in order to maintain homeostasis, a healthy/stable state of the body. PNS is known as the rest and digest mechanism, the activities of the PNS contradicts SNS, it relaxes the heart which slows down the heart rate, lowers stress and decreases blood pressure. SNS and PNS work together to maintain a balance, also known as the sympathovagal balance, allowing humans to be safe and sound or an imbalance would indicate abnormalities associated with the heart (1). Time and frequency domain methods are two of the most common approaches used to accurately assess the function of the ANS (2). Time domain parameters include features such as: (a) standard deviation of NN (normal R-peaks)- intervals (SDNN), (b) square root of the mean of the sum of the squares of differences between successive NN- intervals (RMSSD) and, (c) proportion of the number of NN-interval difference of successive NN- interval which are greater than 50 ms divided by the total number of NN-interval (PNN50) (3). NN intervals were used instead of RR intervals in order to emphasize the use of normal R-peaks. These methods can efficiently analyze HRV through the analysis of the R-R interval which can indicate changes in the HR due to the activities of the SNS or PNS but it's not a sufficient method to discriminate between the SNS and PNS (3). Frequency domain methods such as LF (0.04–0.15 Hz) and HF (0.15–0.4 Hz), LF/HF ratio are often utilized to differentiate between the activity of the SNS and PNS. LF primarily indicates the activity of the SNS but is also partially associated with the activity of the PNS, while HF indicates the activity of the PNS, and their ratio LF/HF is used to determine the sympathovagal balance (3). These indices have made it possible to detect many abnormalities, diseases and possible indication of mortality due to the distorted activity of the heart and the peripheral nervous system. HRV has been used for various applications in research studies which include: analysis of mental and physical stress, classification of drowsiness and other sleep states, analysis of athletic performance and fatigue, studying the correlation between a sedentary lifestyle and mental/physical well-being and analysis of anxiety and depression and various other morbidities associated with reduced HRV.

**Figure 1:**
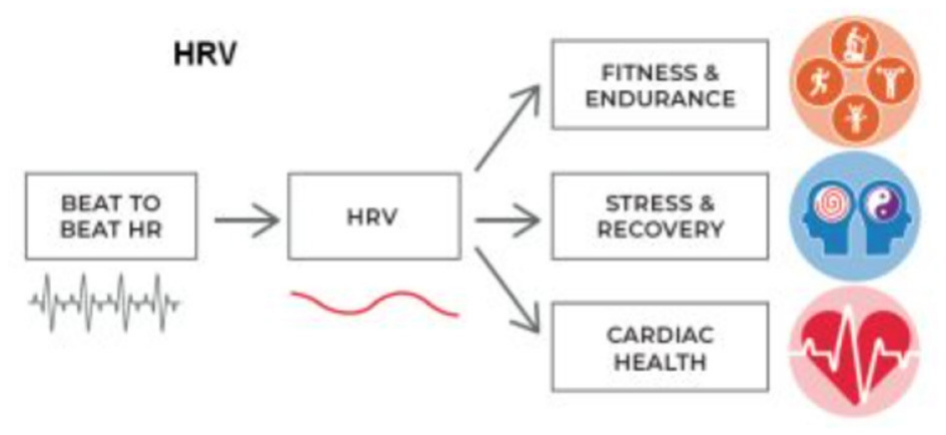
Some important applications of HRV (5).

Kim et al. (4) presented a review paper to analyze HRV and stress, the study described the physiological function associated with stress, as well as HRV related to specific parts of the brain/heart anatomy responsible for the changes associated with stress. The paper presented information related to the anatomy/physiology behind stress, but neglected trends in wearable devices used for data collection, different types of signal processing algorithms used for HRV feature extraction and analysis, machine learning algorithms used for classification of pathologies, wireless monitoring of HRV to improve the health care system and ultimately patient's health and the various applications associated with HRV research (as shown in Figure 1).

This article will analyze the various abnormalities associated with HRV, their detection and analysis using an ECG (electrocardiogram), Respiration, GSR and other wearable devices. The impact of pathologies on the human body and mental state as well as the possible gaps that are associated with each research study.

## 2. Methods

**Figure 2:**
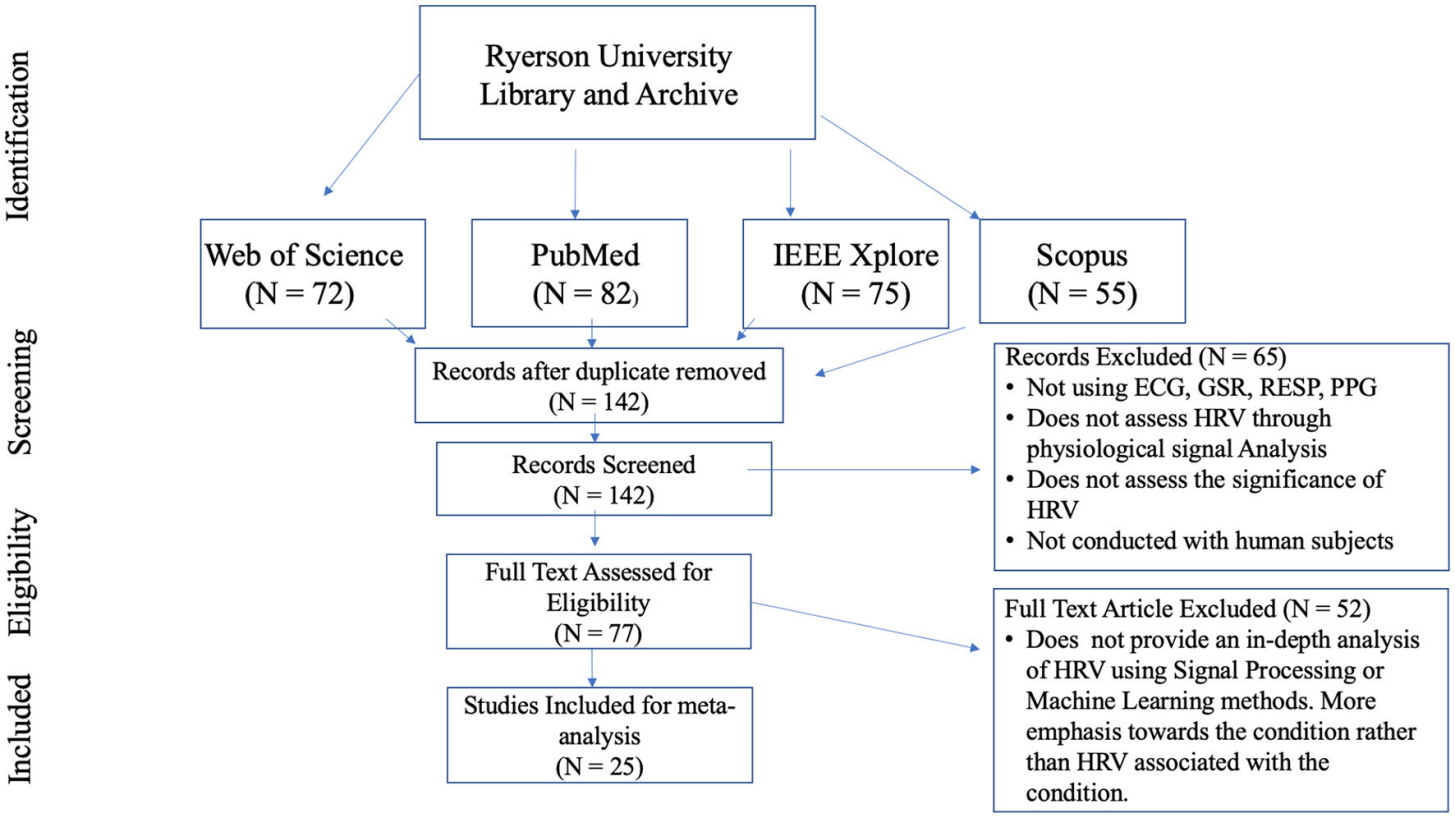
Flow chart for HRV article selections which were used for meta-analysis.

The literature survey was performed through Ryerson University Library and Archives (RULA) online system. PubMed, IEEE Xplore, Web of Science (WoS), Scopus were the primary search databases directed from RULA. The search was allocated toward HRV studies using ECG, EDA, RESP, PPG signal analysis, few papers involved the analysis of EEG or EOG, but were not considered to present information primarily based on ECG, EDA, RESP, and PPG signal analysis. All the reviewed articles were published after 2010 to present information which is not outdated, except one paper which was used to present the function of time and frequency domain analysis. The relevant papers which were reviewed and summarized described the morbid conditions/situation associated with HRV in depth and in detail, any paper which only briefly discussed HRV were not considered. Papers which primarily focused on factors outside of HRV were also not considered. More than 70 papers were reviewed but most of them were not considered for meta-analysis since they did not provide an in-depth analysis of HRV to examine cardiac pathologies, exercise or drowsiness. Accounting for repetitive topics, 18 major concepts were discussed in depth from 25 articles (as shown in Figure 2). The gaps associated with each article were acknowledged and presented.

HRV has a wide range of applications, some of those applications were presented in Table 1. The upcoming sections will scrutinize various research experiments which transpired through the analysis of HRV, investigate the changes within a patient's/subject's HRV due to certain activities and morbidities. It will also examine the void and inconsistency of each research study and outline future direction for HRV research, areas which requires more attention in order to become a more efficient procedure which can have a positive impact on people's lives and prevent chaotic outcomes.

**Table 1:** Research paper associated with HRV detected using an ECG, type of study, results of HRV, concepts being analyzed.

**References**	**Features**	**Application**	**Modality**	**Notable results**	**Method of analysis**
Rosenberg et al. (6)	LF, HF, LF/HF	1D/2D stress study	ECG	2D accuracy 90%	2D scatter plot.
Blood et al. (7)	LF, HF, LF/HF	Depression	ECG	HRV decreases	Frequency Domain
Molina et al. (8)	RMSSD, LF	Posture	ECG	HRV Reduced	Time Domain
Leti and Bricout (9)	RMSSD, LF	Overtraining	ECG	SNS Dominant	Time, Frequency
Walker et al. (10)	SDNN, HF	Noise	ECG	HRV Reduced	Time, Frequency
Wang et al. (11)	R-R, LF/HF	CHF	ECG	100% acc	SVM, KNN
Huang et al. (12)	LF, HF	Anxiety	ECG	HRV Reduced	LF, HF
Pinheiro et al. (13)	LF, SDNN	MI	ECG	HRV Reduced	Frequency Domain
Toni et al. (14)	LF/HF, LF, HF	CVD	ECG	HRV Reduced	Frequency Domain
Shi et al. (15)	HR, SDNN	Emotion	ECG	LF/HF inc	Time, Frequency
Ponnusamy et al. (16)	RMSSD, HF	Seizure	ECG	HRV Reduced	Time, Frequency
Howells et al. (17)	HF	Bipolar	ECG	HRV Reduced	HF
Rios et al. (18)	R-R, RMSSD	Drowsiness	ECG	HRV Inc	Time Domain
Jung et al. (19)	RMSSD, HF	Fatigue	ECG	HRV Reduced	Time, Frequency
Rahim et al. (20)	LF, HF, LF/HF	Drowsiness	ECG, PPG	HRV Reduced	Frequency Domain
Georgiou et al. (21)	RMSSD, HF	Exercise	ECG, PPG	91–99% acc	Time, Frequency
Gontier (22)	LF, HF, LF/HF	Mind Wander	ECG	LF dec	Time, Frequency
Vicente et al. (23)	LF, HF, LF/HF	Drowsiness	ECG	98% spec	LDA
He et al. (24)	ApEn, LF	Stress	ECG	17.3% err	CNN
Schmidt et al. (25)	LF, HF, ST	Stress	ECG, GSR	80% (3 labels)	Adaboost
Cho et al. (26)	SCL, LF/HF	Stress	GSR, PPG	95% acc	KELM NN

## 3. Trends in Heart Rate Variability

In this section, we discuss the trends and evolution of HRV from the oldest upto the most recent research conducted. HRV is not a new topic by any means, initial research on this topic was conducted during the early 1940s. Over the years, along with the significance of HRV analysis, feature extraction and modalities used to assess HRV have also evolved.

**Figure 3:**
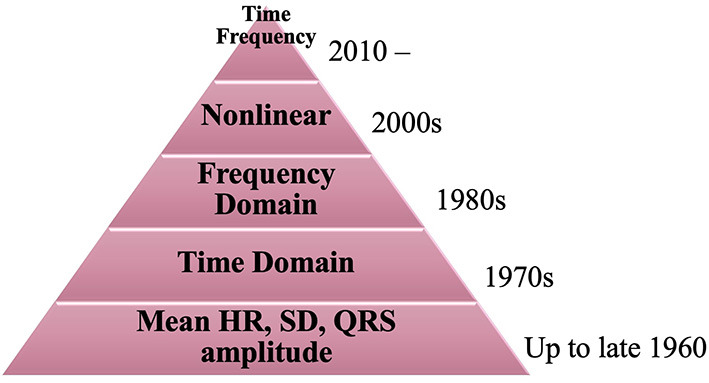
Evolution of feature analysis for HRV from 1940 to 2020.

Features play an important role in discriminating the underlying function associated with any physiological signal. The evolution of features used to analyze HRV is depicted in Figure 2. The earliest feature utilized to analyze HRV was HR from time domain. In 1940, Knox studied the variation in HR due to exercise through mean and standard deviation of each subject's pulse rate (27). This translated to classification of abnormal variability associated with cardiac pathology. In 1958, Simonson studied the amplitude of the QRS complex (28). He derived the mean and SD associated with normal subjects and differentiated them from patients with cardiac pathology. HRV was more distinguishable using animal studies, due to the level of invasiveness allowed for animals. In 1968, Lynch studied the variation in HRV due to shock applied to dogs (29). The data was analyzed using mean and SD of heart rate. A major change occurred around 1969–1970s, R-R intervals were emphasized for their ability to better analyze HRV from ECG which led to the development of time domain features such as RMSSD, pNN50, and SDNN. In 1977, Rompelman et al. presented a literature which compared the various methods used to analyze HRV and demonstrated that R-R intervals were more accurate for measuring HRV in comparison to HR (30). Researchers didn't just stop there, during the 1990s R-R were deemed less effective in comparison to spectral analysis methods. More studies were conducted, which primarily assessed PSD features such as LF, HF and LF/HF associated with ANS impairment due to cardiac pathologies (30, 31). In 2006, Poincaré plots were introduced to present a visual representation of non-linear scatter plots corresponding to cardiac pathologies and reduced HRV (32). Recently joint time-frequency is a recurring trend which is gaining a lot of attention from researchers (2). It is capable of tracking instant changes in HRV through a shorter period, which can effectively diagnose exercise and cardiovascular diseases. Figure 3 depicts the evolution of HRV feature analysis from 1940 to 2020.

**Figure 4:**
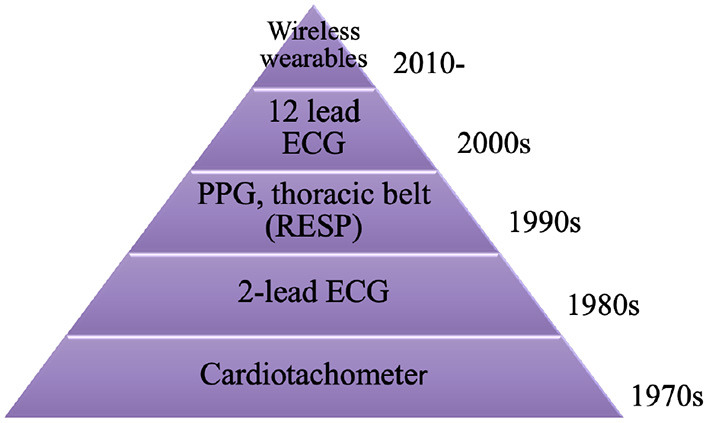
Evolution of medical devices utilized for HRV data acquisition.

Figure 4 delineates the evolution of healthcare devices used to detect physiological signals, which can be analyzed to assess HRV. Data collection is the key ingredient which allows researchers to analyze and detect cardiac pathologies associated with an impaired HRV. Upto the 1980s, cardiotachometer were most commonly used to record a person's electrical signal and record their HR for HRV research (33). Although ECG was developed in 1924, it took about 60 years for them to become affordable for public research. 2 lead ECG's were typically used during the 1980s for HRV research (34). HRV was not just related to heart beat, it also involved blood pressure, mental activity and respiration.

From the 1990s and onwards, HRV research became more diverse. HRV was also analyzed by measuring BP and respiration using PPG and thoracic belt (35). This expanded theories and problems related to impaired HRV, it also added more depth to HRV analysis through information obtained from various physiological signals. Twelve lead ECGs were introduced in 2000, this allowed researchers who were collaborating with clinicians to analyze various cardiac pathologies more effectively (36). The signals obtained were smoother and more efficient in comparison to signals from other ECGs which used fewer electrodes. The current trend involves the use of wearable devices to detect physiological signals, these are much more flexible and portable in comparison to the traditional ECG and PPG devices (25, 37).

**Figure 5:**
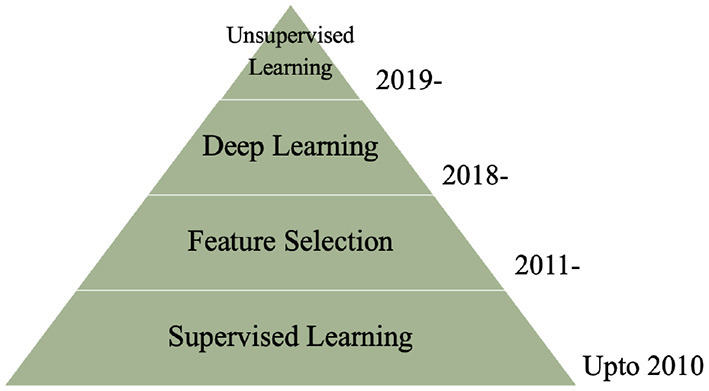
Trends in machine learning for HRV classification.

Figure 5 describes the common techniques used to classify HRV using machine learning algorithms from 2010 to present. Machine learning has been part of many research studies since the mid 2000's. Although it was initially developed in 1950, supervised methods did not become popular until the 2000s. Literature for machine learning was nothing less than an instant success, within the past decade there have been numerous books, literature, research papers, industrial work and health care innovation based on machine learning. It's hard to pinpoint a specific focus in this domain, so we narrowed the timeline to beyond 2010 and focused on common machine learning topics that were the focus for many research conducted on HRV. Supervised learning has been the most common method to classify various cardiac pathologies and symptoms related to HRV since 2010 (37, 38). Supervised models learn the data and predict labels through learned mapping, which allow models such as DT, LDA, and SVM to predict labels based on corresponding features (39). Many research papers in 2011 revolved around identifying the most important features through feature selection algorithms, in order to obtain better classification accuracy and reduce classification time (39, 40). In addition to automatic diagnosis and classification, researchers have implemented shorter windows to extract features associated with physiological function from real-time (41, 42). Deep learning has been utilized more often for HRV research from 2018 to improve automatic classification through real-time. They are capable of detecting hidden patterns from the input through hidden layers, iteratively minimizing errors in data prior to classification. This makes the algorithm more efficient for extracting relevant information related to the topic being analyzed, improves classification accuracy and requires less features for real-time classification (24, 43). An emerging trend on the rise from 2019 is the use of unsupervised deep learning to classify mental stress associated with HRV using autoencoder (44). Self organizing map (SOM) is a dimensional reduction method trained through unsupervised learning, which can indicate the most effective features required to classify stress with high accuracy (26).

## 4. HRV Trends for Data Collection

This sections illustrates the various data collection methods used to detect and analyze HRV. Table 2 reveals the biomedical devices utilized, how they made a significant contribution to the corresponding research and their limitations. Wearable devices are recurrently used in recent HRV research, further indicating the emphasis on remote and wireless monitoring of HRV, in order to make life easier and improve monitoring the health of patients suffering from severe cardiac diseases.

**Table 2:** Data collection methods, their Pros and Cons.

**References**	**Modality**	**Pros**	**Cons**
Rosenberg et al. (6)	Wearable ECG	Detect stress with 90% accuracy	Less effective during pain and non-stationary situations
Blood et al. (7)	Holter ECG	Effectively detect depression and HRV	Accuracy of results
Molina et al. (8)	12-lead ECG	Accurate correlation between HRR and HRV	May cause scar
Leti and Bricout (9)	Polar RS 800	Detect fatigue and HRV in motion	Accuracy of Results
Walker et al. (10)	GE Light ECG	Effectively analyze Noise exposure and HRV	Did not detect correlation between noise and BP
Wang et al. (11)	Wearable ECG	Discriminate between CHF and NSR with 91.3% acc	RMSSD is not accurate
Huang et al. (12)	12-lead ECG	Effectively determine HRV due to stroke and hemodialysis	LF/HF ratio is not accurate
Pinheiro et al. (13)	PTB recorder	Determine prognosis of patients following MI	Cannot deduce causality behind results
Toni et al. (14)	Clickholter ECG	Detect HRV in motion due to antidepressants and exercise	LF/HF, RR are not accurate
Shi et al. (15)	RM6240B ECG	Effectively discriminate between HRV of happiness and sadness	RMSSD, pNN50 and SampEn are not accurate
Howells et al. (17)	MP150 Biopac	Accurately analyzed HRV due to meditation and BD wirelessly	Results lacked most ECG measures
Rios et al. (18)	Gear S, PPG	Possibly recognize drowsiness while in motion	No results were obtained
Jung et al. (19)	ECG sensor	Wireless analysis of HRV due to drowsiness and fatigue	Accuracy of results
Georgiou et al. (21)	ECG,PPG	Analyze HRV with 91-99 % accuracy	Accuracy reduces during motion
Gontier (22)	eMotion Faros	Efficiently detect correlation between awareness and HR	Did not find robust correlations
Vicente et al. (23)	eXim Pro	Detect drowsiness while in motion	Detect drowsiness with 62% sensitivity
He et al. (24)	custom ECG	Detect stress using ulta-short epoch	Accuracy of classification was not revealed
Schmidt et al. (25)	RespiBAN Empatica E4	Detect stress with 93% accuracy	May have resulted from overfitting
Cho et al. (26)	Biopac PPG EDA,UIM	Detect stress with 95% accuracy	Not a viable solution in real-life

### 4.1. Smartphones and HRV

Recent smartphones are more than just a device used for communication and listening to music, these devices include embedded sensors, accelerometers, microphones, digital camera, and various apps based on measuring the affective state (neural, emotion, stress) of an individual. These features allowed researchers to conduct valuable experiments which required wireless monitoring of physiological activity, position, speech patterns, facial expression and affective state, in order to analyze stress levels, behavior and emotion at anytime and anywhere, thus promoting better human health and well-being (45, 46).

Prolonged work periods without sufficient rest/recovery periods can reduce happiness and lead to chronic stress due to mental workload (45). Recent development in technology which integrates artificial intelligence/machine learning (AI/ML) provides insight about a persons stress level at work, during social encounters and sleep. Muaremi et al. (45) utilized smartphones to collect audio, communication and physical activity data during work periods and a wearable Wooho chest belt was used to collect HRV data during sleep. They were able to classify stress using HRV features with only 59% accuracy, indicating that although these advancements are quite fascinating and promotes a healthier lifestyle, it wouldn't be considered effective or rational to use such methods to monitor the health of subjects who are suffering from chronic stress or impaired HRV. The most critical aspect of wearable sensors is their inability to produce accurate data. Utilizing such methods would only seem feasible for empirical studies. They are nowhere near the level required to be effective for use by people suffering from stress or impaired HRV. Smartphones are not designed to promote a healthy lifestyle unlike a wearable ECG sensor, using it for the purpose of diagnosing work stress would require further modification of the design, which would make it more adaptable for health care interventions.

### 4.2. Wearable Devices and HRV

Smartphones and wireless ECG, EEG, and EDA devices would make it possible to detect cardiovascular diseases associated with HRV impairment before it becomes chronic and fatal (46). They make it feasible for health practitioners and people suffering from various cardiovascular diseases (Diebetes, Hypertension) to act proactively and minimize severe outcomes by monitoring their physiological activity throughout the day, including during sleep. Machine learning enable them to predict stress and negative emotions associated with their daily activities, minimizing certain activities may lead to a greater level of productivity and a better sense well-being.

ECG is the most commonly used device with respect to HRV detection (6, 21, 25, 37). Rosenberg et al. (6) utilized a wireless ECG sensor during various situations to measure stress response associated with conference presentations, mental stress test, emergency, and pain. Schmidt et al. (25) utilized Emphatica E4 to measure BVP, EDA, ACC, and TEMP and RespiBAN to detect respiration and ACC (accelerometer). The data collected was used to develop WESAD, a public database which consists of data required to effectively analyze affective states and stress. Cho et al. (26) analyzed HRV, skin conductance (SC)/sweat and skin temperature (SKT) through data collected using a PPG, EDA, and SKT, respectively. They were able to classify stress with high accuracy, using a novel feed forward neural network algorithm and integrated features. Georgiou et al. (21) revealed that wearable devices can detect HRV at rest with 85% accuracy using a PPG and 99% accuracy using an ECG which deteriorates to 85% accuracy during exercise.

Ambulatory detection of HRV is the current resolve for most researchers who hope to make a pragmatic and positive impact on the health and well-being of patients suffering from CVD, hypertension, diabetes, chronic stress and myocardial infarction. Patients suffering from these pathologies need to be monitored throughout the day in order to prevent a serious calamity. Remote monitoring of HRV would undoubtedly benefit senior or chronic patients, who are suffering from cardiovascular diseases but cannot make the effort to visit the hospital all the time, due to the considerable distance and lack of physical ability.

Schmidt et al. (25) were able to classify binary classes of stress by analyzing data collected through wireless sensors with 93.6% accuracy using multinomial logistic regression model. They were able to classify low, mid and high level of stress with 72% accuracy using a random forest algorithm, further demonstrating that chronic stress is hard to predict, although stress can be distinguished from a relaxed state with high efficiency. Cho et al. (26) were able to detect severe stress with wireless PPG, EDA, and SKT sensors from a VR task with 95% accuracy using a kernel based extreme learning machine (K-ELM) algorithm. Although there were numerous studies which classified stress with high accuracies using HRV features, they completely neglected statistical analysis of the data. Machine learning algorithms cannot differentiate between efficient data and errors. They are highly susceptible to biased predictions which arise from biased training datasets, a high classification accuracy can be achieved from erroneous data, if the training data is biased. Physiological signal analysis and statistical analysis can provide an effective corroboration that the data utilized were an efficient representation of a subjects physiological function. Venkatesan et al. (47) developed a novel DENLMS adaptive filter for remote health care applications, in order to remove white noise from ECG signals obtained from patients suffering from cardiac arrhythmia. SVM classifier performed better than other ML algorithms and classified normal/abnormal cardiac arrhythmia with 96% accuracy using HRV features extracted from the preprocessed signal through discrete wavelet transform. Although research is seemingly headed toward the right direction, most wearable ECG devices still require much improvement before they can be used to accurately diagnose heart attack or other cardiovascular diseases. Recent smartwatches did not present accurate information about a subject's HR with respect to their daily life, research studies which used wearable watches to improve weight loss demonstrated that the device produced an ineffective measurement of a person's HR and did not improve weight loss (48). Wearable devices can provide real-time data which can motivate patients to be more careful and promote better self-management in order to prevent chronic outcomes but affordability, adaptability and functionality are still a major concern with wearable devices, especially if they were to be integrated with ML, which poses a major set back and might be the reason that prevents the deployment of such devices. Wearable devices such as a wearable ECG sensor can be utilized to monitor a person's cardiac signal, HR and HRV, which are indicative of chronic outcomes such as myocardial infarction, but they still require further enhancement before they can be considered an effective method for such diagnosis.

### 4.3. Drowsiness and HRV

Around 10–30% of all road crashes are associated with fatigue and drowsy driving. Recent smart watches and portable ECGs are efficiently being utilized to antedate drowsiness, in order to alert the driver prior to any possible accidents. Accelerometer and gyrometer has been examined to assess the users HRV and physical activity, which allows for the detection of drowsiness/fatigue prior to the transition to stage 1 sleep (drowsiness) (18). There is a high correlation between PPG and ECG in terms of detecting HR, Lee et al. proposed a method to automatically remove noise from PPG using a PPG strap which can be used to accurately detect HR while driving, PSD can be utilized to detect HRV in frequency domain, making it a simple and effective method to detect drowsiness through a persons HR (49). Physiological signals such as an ECG have been described as the most accurate representation of drowsiness in comparison to vehicle based method (lane position of the vehicle) and behavioral method (yawning, eye blinking) (49). Although it has yet to be fully established, wireless ECG sensors might be capable of effectively detecting drowsiness, while the driver is driving. In addition, GSM modules can be utilized to send continuous signals to the control room, DC motor can be used to control the speed of the vehicle upon drowsy detection since the driver's reaction would be distorted, LCD can be used to monitor the driver's condition and LED in the rear side of the vehicle can signal the vehicle behind the drowsy vehicle to slow down (50). Roy and Venkatasubramanian (51) proposed a similar idea which involved using an accelerometer to detect motion, SMS to send an alert message to the control room and microcontroller to process the analog signal prior to its analysis through labVIEW and Matlab. Research based on drowsy driving is still relatively new in comparison to myocardial infarction and hypertension which has been studied for over 30 years, which is one of the biggest reasons for lack of adequate research concerning drowsy driving. A reliable and accurate method to detect drowsiness while a person is driving is still a part of ongoing research, it makes sense in theory but HRV is complex and becomes more intricate to detect in motion such as exercise (only 78.6–85% accuracy in frequency domain) and it is especially worse during drowsy driving (21). Vicente et al. (23) conducted a study which involved truck drivers using a drowsy detection detector as well as a sleep deprivation detector and the accuracy of the results were 0.59 and 0.62 sensitivity, respectively. The results indicate that when a truck is in motion, there are a lot of errors associated with wireless ECG detection, some parts of the signal were blank while in motion. Specificity and predictivity were 0.98 and 0.96 using a drowsiness episodes detector and 0.88 and 0.80 using a sleep deprivation detector, disclosing that detection of the signal was the hardest part during this process, specifying drowsiness/awake state upon detection was very accurate through the data analysis of ECG signals using the linear discriminant analysis (LDA) algorithm. The biggest impediment with regards to drowsy detection is the level of interference associated with electrodes. Electrodes are often attached to a person which can hinder their movement, driving requires constant steering to maneuver the vehicle, which produces error and loss of signal detection. Other methods which involve sensors attached to steering wheels are also hindered by the constant placement of both hands on the steering wheel. Most vehicle based measures are deemed unreliable and inaccurate. Most empirical methods that provide partial results which are somewhat indicative of a person's HRV are often imprecise due the lack of control associated with driving, wireless devices still require sensors to be attached to a person which hinders a person's ability to drive and move freely. Smartwatches which are capable of detecting a person's heart rate would be the least intrusive while driving, but would require extensive modification and testing before it could be considered a valid option to prevent drowsy driving. The cost to develop a smartwatch capable of interpreting a person's HRV and drowsiness would be much greater than the current wireless ECG sensors, making it a less likely solution for drowsiness detection which results in thousands of casualties each year.

### 4.4. Video Game and HRV

HCI (human to computer interaction) is one of the various methods utilized for stress analysis, cognitive games such as stroop test are often utilized to assess a subjects ability focus while they are subjected to distraction. Fernandes et al. (52) developed a novel method in order to design a video game FlappyHeartPC which used ECG signals as the input, bridging the gap between human physiology and gaming, such interaction might spark more interest within the user for a boring activity (which is relaxing and beneficial for stress reduction health) such as mediation, fishing, or simply analyzing your physiological signal in a lab. The game design includes a tailor belt worn below the chest with electro-textile electrodes was used as the interface between the sensor and the skin, data acquisition required Bitalino (a specialized data acquisition board), python was used to design the signal processing algorithm used to process/filter the input ECG signal, detect QRS complex and calculate HR. Unity 3D was the engine which made the development of the game possible which can utilize HR as the input for certain physiological analysis (52). The video game is a great innovation which can be utilized for science and excitement but it did not have a specific purpose outside of the gaming business. There have been numerous claims by the gaming industry which proclaims that videos can be utilized to stimulate the brain and improve cognitive abilities associated with memory, reasoning and processing speed. Unlike 2D video games, 3D video games often allow the user to be notably immersed within the virtual environment and absorb more complex information which stimulates the hippocampus. Analyzing just the heart rate alone would not provide sufficient information to analyze an individual's HRV. Python packages can be used to scrutinize the detected ECG signal through time, frequency and non-linear methods but the extracted data may not be accurate enough to validate the users physiological function. However, it can be utilized to improve human health by implementing a stress detection algorithm into the game. If ML learning can be embedded, there are various possibilities with regards to health care applications such as predicting stress and low HRV, which can also antedate cardiovascular diseases.

## 5. HRV Trends for Feature Analysis

### 5.1. HRV and Signal Processing Methods

**Figure 6:**
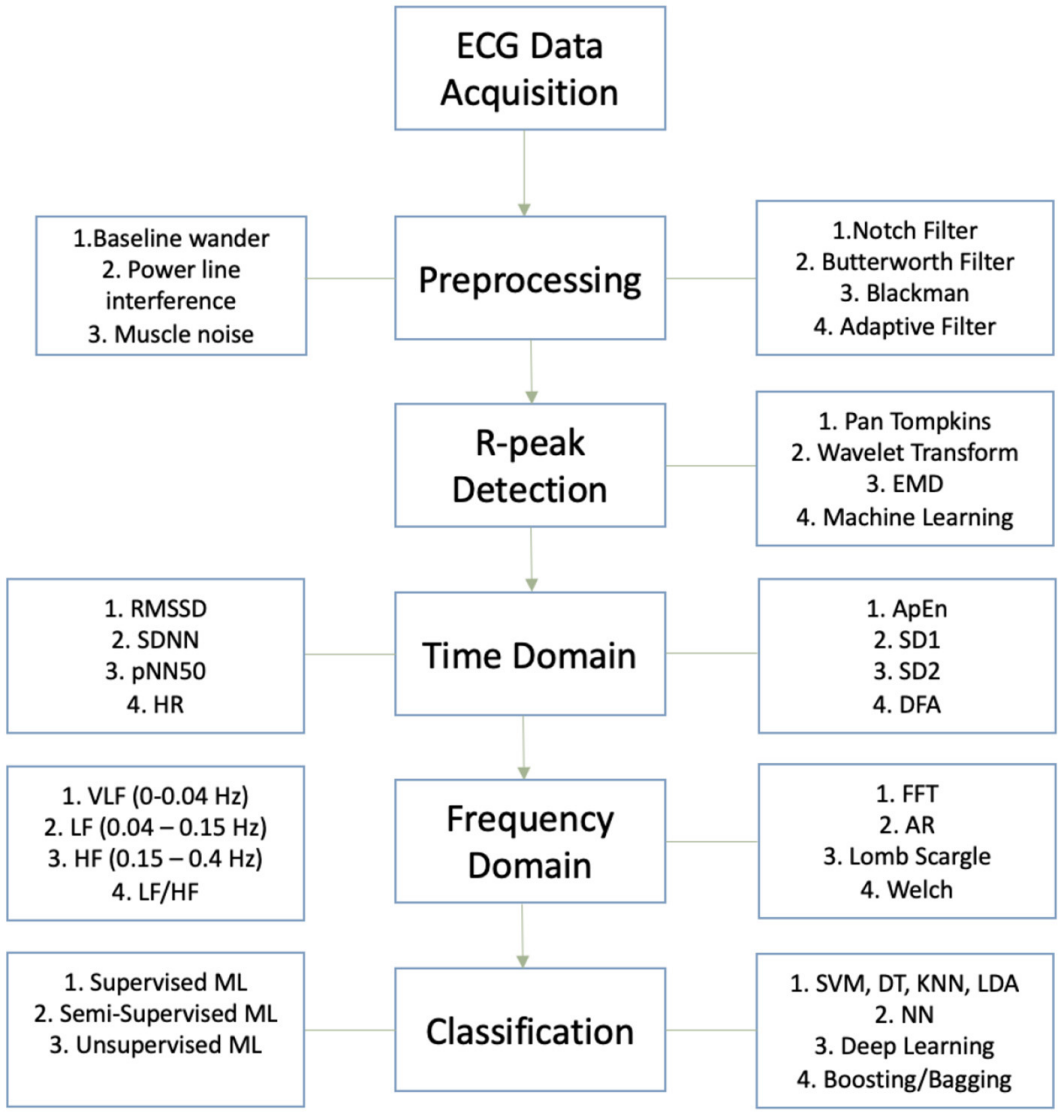
Process flow chart for HRV analysis and classification.

HRV detection is a complex procedure which requires a series of actions, in order to accurately measure the rate of change associated with the R-R interval obtained from the QRS complex, the raw ECG signal first needs to be filtered, processed and reconstructed. Raw ECG signals need to be filtered in order to remove baseline wander, powerline interference and muscle noise (53, 54). After filtering, the ECG signal is a lot smoother and cleaner, which makes it easier to detect the QRS complex. Researchers have developed and innovated many robust R-peak detection algorithms prior to feature extraction such as: Pan-Tompkins alorithm, wavelet transform algorithm and empirical mode decomposition (EMD) algorithm (55–57). Time domain parameters can be extracted using the R-peaks detected but in order to secure frequency domain parameters, spectral transformation of the QRS complex is required through PSD (power spectral density), which can be obtained through Fast Fourier Transform (represents frequency components), Autoregressive (reduces spectral leakages to improve the resolution of the data), Welch Periodogram and Lomb Scargle Periodogram analysis of the QRS complex. Time domain parameters are statistical evaluations of the ECG signal (presents statistical properties) and frequency domain parameters describe how power (variance) is dispersed as a function of frequency (58). Figure 6 demonstrates the process required to extract HRV features from an ECG signal, perform HRV analysis and classify/predict impaired HRV. Table 3 illustrates the time and frequency domain features used to analyze HRV and their correlation to stress.

**Table 3:** Time and frequency domain features.

**Features**	**Description**
HR	The rate of change associated with R-R intervals from HR represents HRV. Increases due to stress
SDNN	The standard deviation of interval between two normal heartbeats (NN). NN measures the total power. Decreases in response to stress. $SDNN=\sqrt{\frac{1}{N-1}\overset{N}{\underset{j=1}{\sum }}{\left(RR_{j}-\bar{RR}\right)}^{2}}$
RMSSD	The root mean square of successive differences between normal heartbeats. Primarily manipulated by PNS activity. $RMSSD=\sqrt{\frac{1}{N-1}\overset{N}{\underset{j=1}{\sum }}{\left(RR_{j+1}-\bar{RR_{j}}\right)}^{2}}$
pNN50	Represents the percentage of the difference associated with NN interval which differ more than 50 ms.It shares a strong correlation with PNS activity, RMSSD, HF
SD1	Non-linear variables derived from the Poincaré plot. Shares a high correlation with HF, RMSSD. Decreases due to stress
SD2	Non-linear variables derived from the Poincaré plot. Shares a high correlation with LF. Increases in response to stress
ApEN	Represents the ratio between SD2 and SD1. Shares a high correlation with LF/HF. Increases due to stress
GSR std	Standard deviation associated with electrodermal activity. Increases during stress
GSR mean	Mean value obtained from measuring the rate of change associated with EDA activity. Increases during stress
Resp Rate	Represents breathing rate, increase in Resp rate leads to increased PNS activity, HF and decreased LF, SNS activity. Increases in response to stress
VLF	Represented within the VLF band (0.0033–0.04 Hz) and it is mediated by SNS activity
LF	Represented through 0.04–0.15 Hz within the PSD, it is mostly used to indicate SNS activity but can specify PNS activity
HF	Represented by the frequency range of 0.15–0.40 Hz and solely indicates PNS activity
LF/HF	Represents ANS activity, increases in response to increased stress and decreased HRV

### 5.2. HRV and Stress

**Figure 7:**
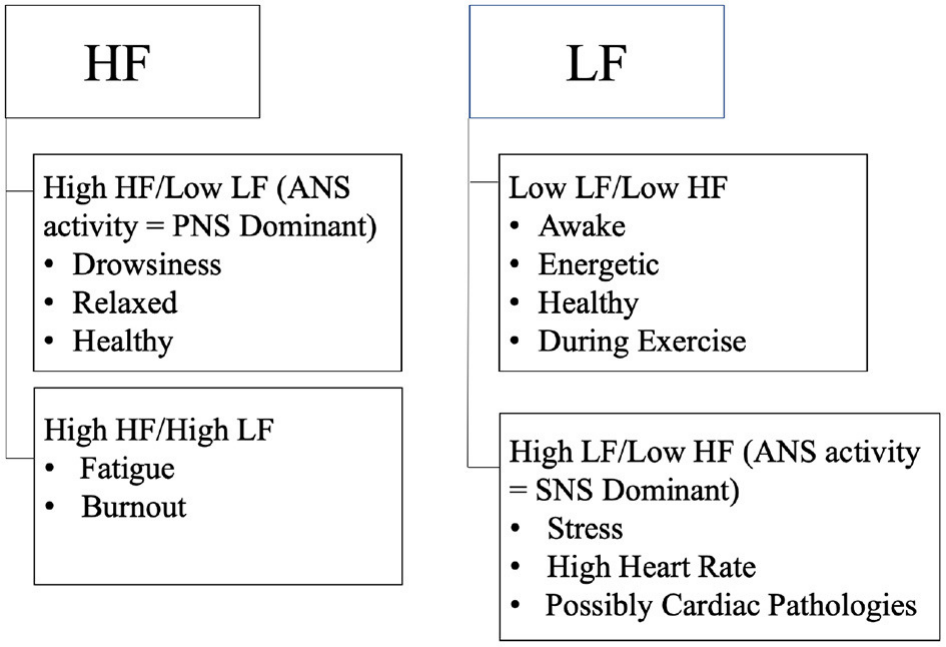
Describes LF and HF associated with stress, drowsiness, awake, and fatigue.

As described in Figure 7, stress is primarily associated with the activity of the SNS, increased LF (0.04–0.15 Hz) band in frequency domain and reduced HRV. It activates due to perceived danger (such as a deadline, financial worries, exam) and increase in cortisol levels causing the activation of SNS which mobilizes the body's activity under stress in order to react/respond rapidly to any dangerous situations (6). Rosenberg et al. (6) analyzed the levels of stress in response to various situations including public speaking, math, exercise, mediation, pain and cognitive tests. ECG signal obtained through a wireless ECG sensor was processed to measure HR, time and frequency domain features such as SDNN, PNN50, RMSSD, LFn, HFn, LFp, HFp, LFiA, HFiA. LF/HF (normalized, power, instantaneous) were extracted to measure HRV as well as SNS and PNS activity associated with HRV. There are different levels of stress depending on the person's HRV, most often 1D frequency domain methods such as sympathovagal balance (LF/HF ratio) were used since they are more efficient/ simpler than the time domain methods (RMSSD, PNN50, SDNN), which takes longer to assess, although the efficiency of the method can be significantly improved (6, 23). Rosenberg et al. used a 2D scatter plot (LF Vs. HF on a 2D scatter) using multiple variables such as LFn, LFn, LFp, HFp, LFiA, HFiA and their ratio and compared it with the 1D methods (LF/HF ratio or LF, HF computed independently) for different stress tests such as: mental stress, pain, emergency, meditation, and pain. The results concluded that 2D scatter plots were much more efficient than 1D univariate methods, 2D results produced accuracy of 90% or above, whereas 1D methods were around 70%. 1D variables are very linear, unlike stress, they cannot effectively discriminate between 2 tests (such as: Math and exercise) that lead to similar heart rates. However, 2D scatter plots can efficiently differentiate between each ANS activity due to the different activities, resulting in much more efficient results and categorization of ANS activities (SNS and PNS activity) due to different stress states. The accuracy of the experiment is questionable since only 10 participants were used, which is less indicative of the overall population, one individual can have distinct patterns which is not comparable to the rest of the world during exercise or math. Another questionable result would be the result of the HR, exercise should result in a higher HR since the heart starts pumping faster to pump blood to the rest of the body during exercise, in order to match the incremental demand of the exercise, resulting in an increased HR upto 5 min post exercise. LF value during exercise was also rather low, exercise promotes efficient use of one's energy allowing an individual to be more awake/alert throughout the day, which is more associated with the activity within the LF band. A 3D assessment which includes time would probably result in a more comprehensive analysis, effectively specifying the periods associated with increased levels of stress.

### 5.3. Short-Term Signal Analysis and HRV

Rosenberg et al. (6) have also indicated that time of the epoch used to assess HRV in time domain is very important, 3 min is the minimum epoch that can be used by RMSSD in order to measure fatigue in athletes, but 5 min epoch are optimal for stress analysis, otherwise preprocessing the signal may lead to filtering out valuable information which would result in inadequate, less efficient output and representation of HRV activity associated with stress. Castaldo et al. (41) analyzed HRV using ultra-short term HRV features in order to assess mental stress in real-time. Theoretically stress is generally associated with perception, it can be due to internal perception such as negative emotions of anger, anxiety, fear, depression and mood swings or it may be induced by external perception of the world around us, such as an upcoming exam, presentation or deadline which causes us to worry, lose sleep and accumulate stress (37). MeanNN, stdHR, HF features resulted in the best accuracy when classified through an automated classifier such as TPOT, which classified HRV using various ML models (SVP, MLP, neighbor search IBK, C4.5, and LDA) and indicated which algorithm was able to classify stress with the highest accuracy (41). Statistical testing is an essential component of every research study, in order to verify, validate and understand the significance of the results obtained. Statistical hypothesis testing legitimizes the efficiency of the results and encourages further expansion of notable methods which can have a significant impact on people suffering from drowsiness, impaired HRV, and cardiovascular diseases (59). Current trends in machine learning hints that there is a bigger initiative for real-time analysis, various algorithms were developed to permit real-time analysis using ultra-short term epochs of 3 min and under (60). In certain cases even 1 min epoch can produce data which can be analyzed to effectively classify HRV using specific features, some features are peripheral, by reducing such features, HRV can be classified in real-time and with higher accuracy (41). Most PSD methods such as FFT, Lomb Scargle periodogram and Autocorrelation are capable of producing useful results which can be used to detect HRV from only 3 min, but it requires the subject to be stationary and stable. Experiments which involve motion (e.g., exercise, driving) produce erroneous results. Most research studies emphasized the use of time domain features to analyse HRV from short-term durations, which is also simpler to extract than frequency domain features. Time domain features are not consistent and often vary, which makes HRV analysis very complicated and flawed. Frequency domain features are more accurate in comparison to time domain methods but do not produce valid data from shorter windows since the rate of change associated with R-R intervals are being compromised as well. Short-term duration does not allow the data to fully grasp the activity of the heart, HRV is derived from the rate of change due to fluctuations in HR, shorter windows produce less data and less accurate results. Short-term duration results in minimizing most of the data which also removes valuable information needed to understand the overall condition of the subject (2, 61). Pre-processing is also limited by short-term durations since most of the data might be filtered out if the data is noisy, which is reasonable from subjects under stress. Short-term data can make a significant contribution to the health of patients suffering from CVD, by allowing them to monitor their heart rate in real-time from a distance using ambulatory ECG sensors but extensive research is needed to find viable solutions which can minimize the motion artifacts and reduce errors.

### 5.4. Low/Reduced HRV

Lower/reduced HRV transpire as a result of increased SNS activity and reduced PNS. It often infers that higher HR/blood pressure leads to various morbidities and increases the chances of mortality. HRV of patients/subjects suffering from depression is very low, VLF (0.003–0.04 Hz) has been positively associated with depression and it is also one of the strongest indicators of depression (7). Blood et al. were able to make these diagnoses using correlation analysis (scatter plots), which compares the activity of the LF, VLF, and HF due to various symptoms associated with depression. The research study also revealed that low HF (equivalent to low HRV) emanate more anger, sadness, peer problems, and anxiety, while decreased VLF would cause the development of chronic inflammation, and dysregulation of VLF (associated with metabolic process, thermoregulation, renin angiotensin, regulates blood pressure and fluid balance) which would result in more fatigue and depression (7, 27, 62). The research neglected any possible solution to counteract depression, wireless sensors can be incorporated into biofeedback systems in order to monitor a person's HRV and provide feedback to improve their emotional well-being by increasing their HRV. Nexus has developed biofeedback devices which are capable of measuring physiological activity associated with impaired HRV and providing solutions to improve their physiological function. Mendi developed a biofeedback device to strengthen cognitive function associated with low HRV and stress, which can improve depressive symptoms as well. Interaxon also developed a biofeedback device the muse to counteract low HRV and stress through guided meditation. These devices are expensive and would not be considered as a cure for chronic conditions such myocardial infarction but they can improve depressive symptoms which is often associated with prolonged stress and imbalanced physiological parameters associated with impaired ANS activity. Reduced HRV is a risk predictor of heart failure after acute myocardial infarction, a warning sign for diabetic neuropathy, and has been associated with patient suffering from sleep apnea, dilated cardiomyopathy, fetal distress as well as congestive heart failure (11). Decrease in HRV is correlated to reduced SDNN and a shorter R-R interval. Significantly lower LF along with a reduced HRV antedates sudden cardiac death for patients suffering from CHF, due to the impaired activity of the ANS, which is unable to respond/react accordingly to the treacherous situation. Both time and frequency domain variables (such as SDNN, LFn, HFn, LF/HF) were used as predictor of morbidity/mortality within the study conducted by Wang et al. (11). Moreover lower HRV and vagal tone indicated through low HF, shorter R-R interval and smaller RMSSD values are associated with epileptic seizure (16). A study conducted by Shiro et al. analyzed the correlation between HRV, chronic neck pain and shoulder pain specifically within females (63). Common cause of neck and shoulder pain is repetitive/over work which can cause an increase of intramuscular glutamate and lactate within the traps. Isometric contraction was performed to indicate the effect of muscle load, LF/HF was lower (increased HRV) within the relaxed and pain free subject but it was inactive for the pain group which was attestation of impaired ANS activity (63). Inactivity of LF/HF is not a clear and concise representation of ANS dysfunction, 2D scatter plots may have provided more efficient results. Undetected signals can also produce dormant results, ECG sensors are not as competent when monitoring subjects in motion. Interpolation is capable of estimating rational values which can be used to fill in the missing values. The results would not be perfect but it may produce frequency domain values which can reveal the most likely outcome due to neck and shoulder pain. A research study analyzed HRV due to fatigue, in order to prevent athlete performance burnout and overtraining (9). Competition has been associated with increased LF/HF and SNS dominance, indicating that athlete's may suffer from more fatigue, stress and anxiety during competition (64, 65). Studies revealed that HRV and HF decrease with an increase in age (9). Aerobic training positively impacts HRV and HF, which was indicated through the positive correlation with time domain parameters such as SDNN and RMSSD and HF. Excessive training can cause impairment of the cardiovascular control system, negatively impact a competitors mood/state which has been associated with injury and fatigue, resulting in reduced HRV and HF. Increased SNS activity which is specified through an increase in LF, compensates for reduced cardiac performance and helps recover normal blood flow. High SNS is also associated with fatigue during training which correlates to reduced HRV and HF (64, 65). Two days after the competition, an increased HF suggested a rise in PNS activity and HRV, disseminating that exercise/training improves vagal tone and helps to maintain ANS modulation (64). Unlike Fourier transform which neglects the time-localization information, wavelet transform extract information with respect to time and frequency, which is excellent to detect HRV information which is not stationary. It can detect the instantaneous change associated with HR due to exercise more efficiently than common PSD methods such as fft and AR periodogram which is more effective for frequency domain analysis and stationary processes (2). Missing data and ECG signal recording inactivity is a common problem associated with monitoring HRV in motion and during exercise. Interpolation, reconstruction of large gaps and reconstruction with localized estimation are few methods which can help rectify the data and extract feasible frequency domain features (66). There is a higher probability/occurrence of myocardial infarction associated with older women as a result of lower HRV and ANS dysfunction (13). HRV analysis also revealed that SDNN, RMSSD, triangular index were significantly worse for women than men, additionally reduced HRV is the strongest predictor of myocardial infarction (13, 67). Resting HR is a robust indicator of myocardial infarction and coronary death within women, low HR as well as increased HR associated with depression antedates coronary artery disease. Women and men require different treatments for an accurate prognosis due to sexual dimorphism associated with men and women. Time domain methods are not capable of differentiating between SNS and PNS activity which can make data analysis somewhat biased and based on preconceived assumptions. Statistical t-test or chi squared tests can corroborate the plausibility of the data and help determine whether the results presented are statistically significant (3). Patients suffering from stroke and requiring hemodialysis also indicated a lower HRV, post dialysis presented an increased VLF, LF, TP, and LF/HF ratio (12). VLF is robust in terms of prognosis for CHF. Lower HRV is also associated with adverse cardiac states, increased morbidity and mortality within patients suffering from ESRD (end stage renal disease). Relaxing music such as classical music improved HRV in patients with cardiovascular dysfunction and dementia. Interestingly classical music at high intensity also reduced HRV, although sufficient analysis was not provided. LF was reduced during heavy metal which may indicate that it is harmful and causes increased fatigue. Higher intensity of music increased sympathetic tone on HR, the reaction designate that music is perceived as a threat by the ANS and may induce stress/fatigue (68). The frequency domain data was analyzed via FFT algorithm which is capable of producing miscellaneous results due to its inability to apprehend transient signals through unspecified capture windows. Specific ranges within the capture windows are capable of producing valid results depending on the duration of the transient signal, otherwise it can result in data leakage which distorts the feature values obtained. Bandwidth filtering of the signal was not mentioned, which can lead to aliasing and result in incorrect frequency and amplitude. Do Amaral et al. (68) identified that music can increase or reduce HRV based on the type of music and its impact on HRV. Music therapy involving soothing music improves HR, it has been utilized to improve cardiac function after taking cardiotoxic medication (68, 69). Heavy metal and metal rock reduced HRV and the modulation of the heart indicated through reduced SDNN. Although SDNN is capable of interpreting the overall HRV, it can increase or decrease as a result of decrease in HRV. Its simple to compute but does not provide sufficient information to understand ANS activity associated with reduced HRV (3). Kubios was used to analyze the data, its a software which automatically produces results in time and frequency domain. It uses automatic filters which are likely to produce imprecise results if the signal is very noisy (21).

## 6. HRV Trends using Machine Learning

This section discusses the recent studies which classified HRV using machine learning algorithms. Table 4 demonstrates the accuracy achieved and the computational cost associated each machine learning algorithm. In order to make a significant impact and connect to as many patients as possible, remote monitoring and analysis of HRV needs to improve. Machine learning is revolutionizing society. It is progressing at a very fast rate to make remote monitoring of HRV effective and accessible to everyone. HRV analysis through machine learning is creating a major impact in research and the world at large, making it possible to accurately antedate diseases, lower healthcare cost and help patients make the right decision, with regards to treatments and therapies.

**Table 4:** Recent publications based on HRV + Machine Learning. The accuracy produced and the theoretical computational cost required by the algorithm.

**References**	**Accuracy (%)**	**Computational cost**	**ML algorithm(s)**
Castaldo et al. (41)	94,88,94,94	0(*n*),0(*kd*),0(*n*log*n*),0(*nd*^2^)	MLP, SVM, C4.5,LDA
Cho et al. (70)	90.19	0(*n*·*k*·*d*)	CNN
Cho et al. (26)	95	0(*n*^4^)	K-ELM
Coutts et al. (71)	83	0(*W*) *W* = 4*IH*+4*H*^2^+3*H*+*HK*	LTSM
Taye et al. (72)	98.6	0(*W*) *W* = *IH*+*HK*	ANN
Arsalan et al. (73)	92.85	0(*n*)	MLP
Lima et al. (38)	80	0(n*log(*n*)*d*k)	Random Forest
Kublanov et al. (74)	91.3,87.8, 87.1,88.2	0(*nd*^2^),0(*kd*), 0(n*log(*n*)*d), 0(c*d)	LDA,SVM,DT,NB
Ma et al. (75)	96.58, 98.2	0(*n*·*k*·*d*), 0(*n*)	CNN, MLP
Persson et al. (76)	77.5,83.4, 82.4,85.4	0(*nd*),0(*n*^2^), 0(*nt*),0(n*log(*n*)*d*k)	KNN, SVM, AdaBoost, RF

### 6.1. Stress Classification Through HRV Analysis

Alhitary et al. (37) have indicated that people need a little bit of stress in their life to stay focused, alert and energetic, so that they can solve the problems they face in their daily life. Alhitary et al. (37) also revealed that if people let stress linger around and continue to worry, it can evolve into chronic stress, leading to more anxiety, lack of coordination and reduced level of productivity. If stress is not detected early, it often leads to many heart related diseases such as hypertension and CVD. In addition to increasing the chance of an infection, it is also a major cause of emotional trauma such as depression. Schmidt et al. (25) developed WESAD, a multimodal public dataset using wearble devices, which includes data for stress and affective emotions. They detected the affective states of users through Emphatic machines such as RespiBAN and Empatica E4, which was placed on their chest and wrist, respectively, to assess their neural state (baseline brain activity), stress levels and amusement condition (emotional state, in this scenario humor was induced). Utilizing the machine learning classification algorithm Adaboost, they were able to classify stress/no stress conditions with 93% accuracy using features obtained from physiological signals (e.g., ECG, EDA, Respiration, skin temperature, accelerometer). Adaboost is a boosting classifier which is considered a strong learner, it is made up of cascade of weak learners such as DT. Unlike weak learners, boosting models learn from the training data and iteratively reduce error by adding a weak learner based on the weight associated with the error. It can predict labels with high precision, by adapting to the training data and minimizing errors. It takes longer to train adaboost and it is not effective for learning imbalanced training data (77).

### 6.2. HRV Analysis Using Random Forest

**Figure 8:**
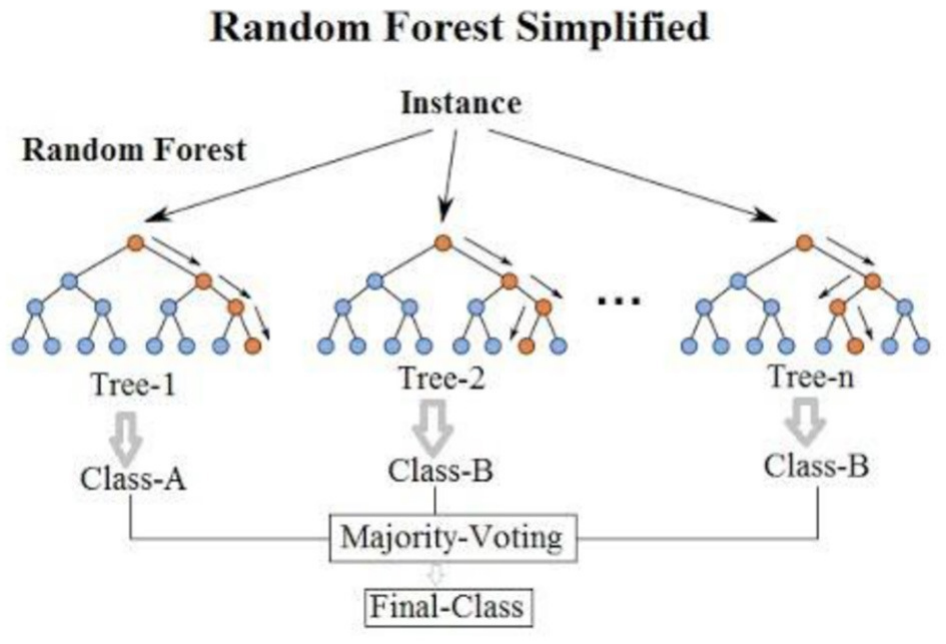
A standard classification process for a RF algorithm. Source: Montantes (78).

Lima et al. (38) revealed that research experiments are sometimes unpredictable as LF and LF/HF activity during stress decreased for certain circumstances where stress was detected. Delineating the changes in ANS activity plays a significant role toward preventing CVD and stress. ANS is regulated by the CNS, it comprises multiple neuroanatomical structures. CNS sends a signal to the SA node in order to adjust to physiological arousal, it's also responsible for responding and adapting to environmental changes (38). The structures of the brain influences the activity of the heart. In contrast to the theory that SNS activity increases during stress, LFnu decreased for some subjects during instances of stress. In order to efficiently classify stress and detect the event, they implemented a SVM algorithm which included an optimal hyperplane to separate subjects whose LFnu increased and decreased during stress (38). There was also a contradictory decrease in LF, LF/HF ratio during stress phases. Using time domain HRV features such as: HR, RR-interval and SD1/SD2, they were able to classify stress with 80% accuracy through Random forest (RF) classifier. SCL, SCR and rise time extracted from EDA resulted in 77% accuracy using RF. Stress labels were obtained by comparing the results to a baseline for both experiments (38). These features used to predict stress are not consistent with the theories associated with ANS activity, stress was classified by comparing the results to a baseline signal and HR which always varies was a prominent predictor of stress in this scenario. Classification report which includes TN, TP, FN, FP accuracy behind stress detection would better indicate the reason behind the contradictory results, which varies from standard theories associated with ANS activity (such as: a decrease in contrast to an increase in LF, LF/HF ratio during times of stress). RF is a bagging algorithm which also implements an ensemble of decision trees much like Adaboost. In contrast to most strong learners which are prone to overfitting and memorizing the data, bagging algorithms reduce variance in a data which improves accuracy and reduces overfitting. Most models perform more effectively if features with linear pattern are utilized, RF is a curve based algorithm which can efficiently adapt to non-linear parameters. It also requires a longer training period and a lot of computational power to handle the excessive number of decision trees used (A standard classification process for a RF algorithm is shown in Figure 8).

### 6.3. Classifying HRV From ECG and EDA Features Through ML Classification Algorithms

Posada and Bolkhovsky (79) conducted a study to assess Psychomotor vigilance (PVT-measures reaction time), auditory working memory (n-back task), visual search (ship task) through ECG, EDA features, and ML classification algorithms. Lack of sleep due to stress reduces vigilance and the ability of working memory regresses with prolonged lack of sleep. The detection of the activities indicated that PVT, auditory working memory and ship search all had different effects on ANS. SCL, TVsym, and LFn which are SNS biomarkers were the most significant differences associated with EDA and ECG activity during each task. Data was classified using linear kNN, linear SVM, LDA with 66, 66, and 62% classification accuracies, PVT along with ship search was classified with 69% classification accuracy using kNN, while working memory was classified with 69% accuracy using LSVM. The study was conducted upto 24 h, classification after 20 h indicates that ANS activity diminished after 20 h of wakefulness, but surprisingly recovered after 24 h (79). In order to improve the low classification accuracy, feature selection would be an appropriate method to reduce the number of features which are futile. Dimensional reduction methods such as PCA can also be used to classify the data with the most valuable features, which can also reduce model complexity, improve classification accuracy and reduce overfitting (80). Training data is almost of no importance for KNN algorithms, it is an instance based algorithm which cannot derive any discriminative function from the training data, large number of features makes it difficult for the algorithm to derive the distance between each dimension, which also results in a low accuracy. Noisy data-set also hinders performance, outliers and missing data have to be optimized to improve performance. Noisy data also negatively impacts SVM, making feature engineering an essential component to improve performance (81). Noise can produce flawed data which is random and is not normally distributed, if the data set is non-gaussian, it negatively impacts LDA algorithms ability to preserve the complex structure data needed for an efficient classification. Data wrangling is often utilized prior to training/testing a dataset, to minimize outliers, missing data and transform the data-set in order to make it more appropriate, which would make it more efficient and effective for classification using unsupervised models (82). There is a recurring trend between low classification accuracy and irrelevant features, although more data may improve classification accuracy, the appropriate feature selection method is capable of significantly improving the efficiency of the results (83). Ideally more features result in better accuracy, but Taye et al. (72) demonstrated that innovating features based on the specific domain is a much more efficient approach. They were able to reduce 7 dimensions and improve classification accuracy by 26.6% using a novel QRS complex feature engineering method. This is another example of reducing the computational costs while improving the efficiency of the methods. Additional research which combines such methods with wearable devices will allow researchers to dive deeper and further reduce the gap which prevents remote monitoring and diagnosis of HRV from being accessible to everyone in today's healthcare. COVID-19 has really addressed an urgent need for remote health solutions, researchers can revolutionize healthcare by combining ML with HRV in order to reduce stress and cardiac pathologies.

### 6.4. HRV Associated With Affective Computing, Classified Through NN and SVM

Mobile devices which can monitor health accurately can positively impact a large population of people. This research is targeting more than just CVD and stress, it is expanding to cancer detection, muscle injuries, circadian rhythm and affective emotion (emotion, stress due to age and gender). Rukavina et al. (84) analyzed physiological signals obtained through EMG, EDA, ECG and respiration to distinguish between various affective states based on gender and age. NN and SVM reported the highest classification accuracy using features Mean, Std, fEMG, low valence low arousal (LVLA), low valence high arousal (LVHA), high valence low arousal (HVLA), high valence high arousal (HVHA), and neutral. Mean and std were analyzed to detect skin conductance associated with SNS activity. Valence and arousal state were scrutinized by studying the correlation between neural states and emotions. Performance was evaluated using the leave one out cross validation (LOOCV) method. The classification accuracy was blunted by a small dataset, which can be improved through more trials and additional features (84, 85).

Pathoumvanh et al. (86) revealed that ECG biometrics are different from affective states, they were able to classify HRV conditions with 97% classification accuracy and also achieved 80% robustness study accuracy, using only a single beat ECG feature and LDA algorithm. LDA is a simple model that predicts labels based on the highest probability obtained through Bayes theorem. Fisher's linear discriminant analysis is an extension of LDA which can reduce RMS dimensions and classify data with higher precision. Unlike DT, it's not prone to overfitting (87).

### 6.5. Stress Induced Through VR Environment and Classified Using Extreme Learning Machine (ELM)

**Figure 9:**
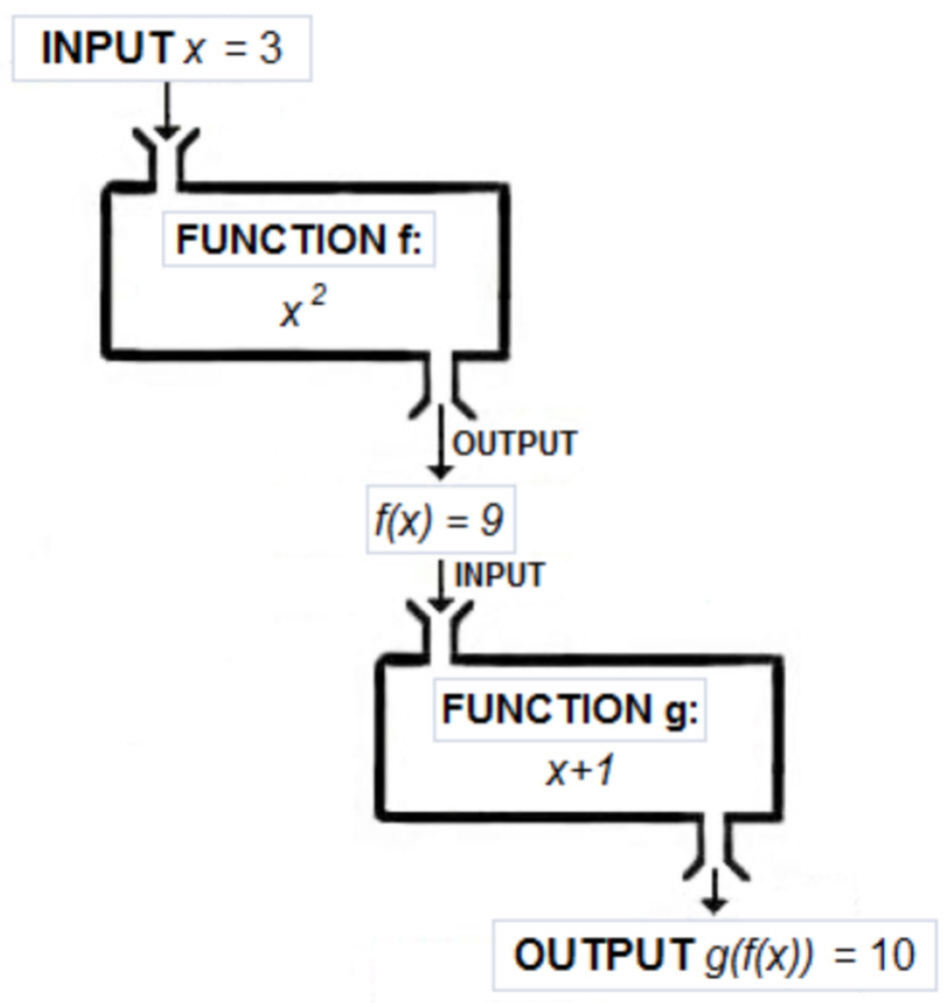
Process of transforming data in a deep learning ELM algorithm. Source: Terry-Jack (88).

Cho et al. (26) were able to classify stress with 95% accuracy using features obtained from three physiological signals (PPG, ECG, EDA) through Kernel based Extreme Learning Machine (K-ELM). K-ELM is based on a single hidden layer feedforward neural network which generates input weights and hidden layer biases, it requires less resources to classify results with high accuracy and leave one out cross validation (LOOCV) was used to evaluate the classifier. KELM is capable of discriminating between classes with high efficiency due to its ability to transform data which is hard to distinguish into linearly separable data while utilizing specific features (as shown in Figure 9). However, the features are selected randomly without utilizing an established algorithm like CNN, which makes the results unreliable and random for a specific dataset. The algorithm might not effectively classify other data-set as efficiently. LOOCV takes advantage of one feature to evaluate model performance, it has a high variability despite classifying labels with high accuracy. LOOCV also requires a lot of time to fit and evaluate the data. The experiment unfolds the possibilities which exist for wireless monitoring of stress, accurate results produced from HRV through a wireless device is an indication of phenomenal solution that is yet to be produced in health care due to the lack of efficiency, this is an indication of many possibilities that may arise within the next decade for wireless monitoring of HRV and human health through the use of machine learning and wearable devices.

### 6.6. Convolutional Neural Network (CNN) Used to Detect Stress Through HRV

**Figure 10:**
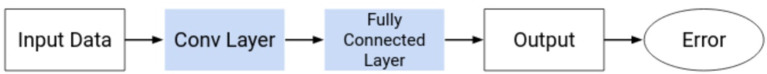
A typical CNN architecture for stress classification using HRV parameters.

**Figure 11:**
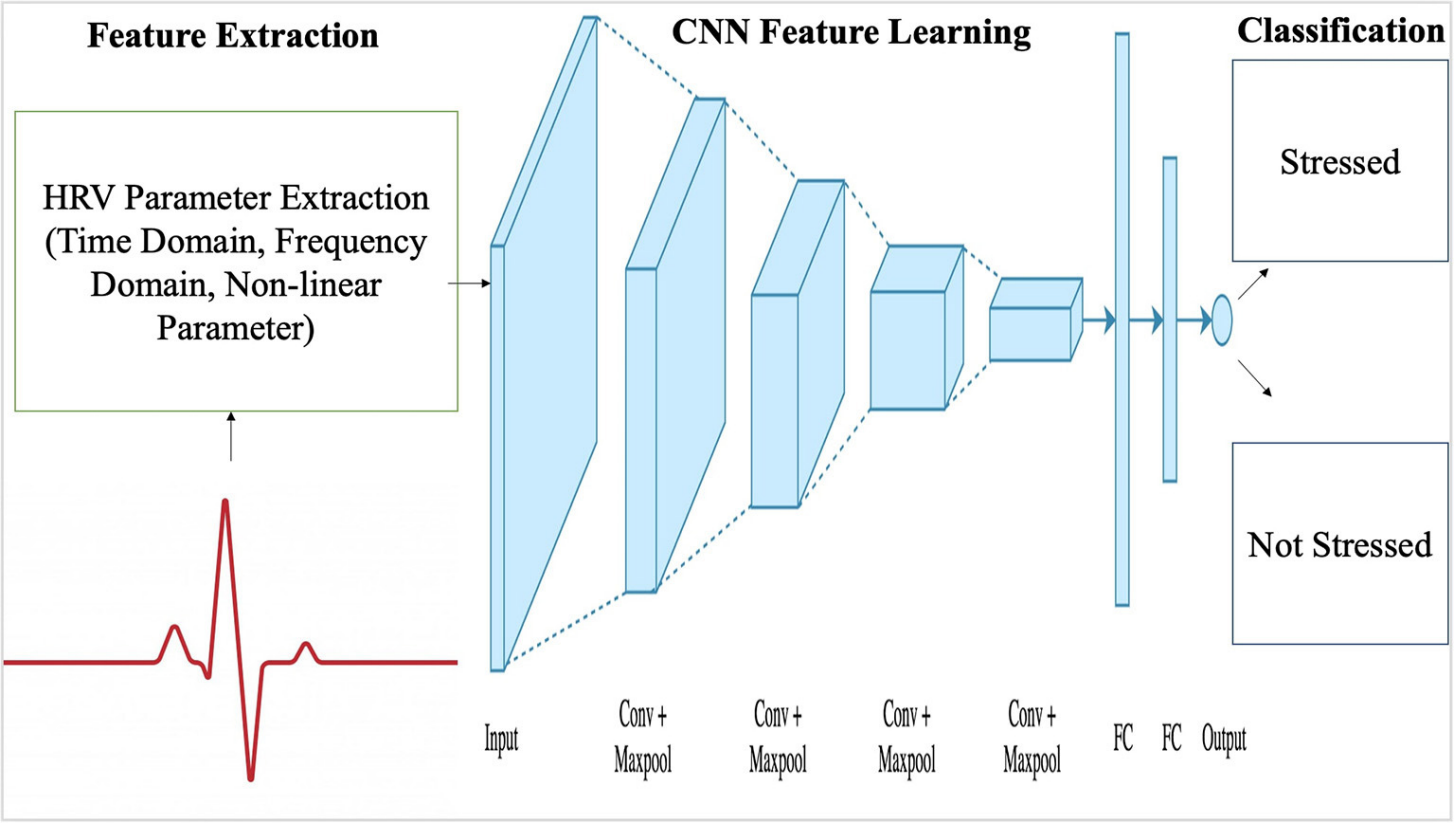
An example of a CNN algorithm process flow chart. Source: Aishwarya (89).

Whether it involves stress, CVD or drowsiness detection, one of the limiting factor that exists within most innovations is their inability to perform during real-time applications. He et al. (24) was able to classify cognitive stress using features which were observed in real-time through ultra short 10 s windows. They utilized Lomb scargle periodogram to obtain the PSD from the detected R-peaks. CNN was used to understand the 0.04–20 Hz band from the PSD and extract the relevant features from the input layer. CNN utilizes automatic feature learning for fast and accurate analysis of cognitive stress through HRV features. CNN is similar to other deep learning methods, but it also consists of a convolutional layer in its hidden layer (process flow chart shown in Figure 10). It can automatically capture the relevant information from the input unlike other feedforward neural networks, it can reduce the image features to the point where the information becomes very simple to process without losing valuable features required to make an accurate prediction. A typical architecture for HRV classification using a CNN algorithm is shown in Figure 11. In order to classify stress using data from the PSD, 10 layers were utilized which included an input layer of size 799 ×1 ×1, a convolutional layer that consisted of 6 filters with size 4 ×1 ×1, batch layer, RELU layer, dropout layer, 3 fully connected layers with batch normalization between them, softmax layer and an output layer. Batch normalization layer normalizes the data, reduces overfitting, and allows each layer to learn independently. RELU layer is essential for effectively updating the data with each iteration. Dropout layer is used to reduce overfitting. Fully connected layers connect the information obtained after being filtered with the output later, in order to classify the data. Softmax layer allows for multiclass classification of the data. CNN produced a 17.3% error rate, which was 7.2 and 32.6% lower than SVM, using comB (combined) feature and LF/HF ratio, respectively. CNN performed better than conventional methods in terms of ER and FAR (false acceptance rate) (24). CNN is really an extension of deep learning models which only use fully connected hidden layers, it's more effective due to its ability to reduce errors through the convolutional layer. Unlike most deep learning models, the convolutional layer allows the model to adapt to the input data more effectively, the activation depth significantly improves due the number of filters, resulting in better classification (43). One of the biggest advantages of CNN is its ability to predict labels with high accuracy using less features than standard deep learning models. Overfitting is the downside to all deep learning models, batch size and epochs allow the model to update the weight and minimize error, but such a method is also prone to overfitting especially if its a smaller dataset. The development of CNN has made remote monitoring of HRV much more effective and simpler. CNN is a powerful algorithm which can be used to extract valuable features from raw ECG signals obtained through a wireless ECG sensor, and classify HRV and stress with a high accuracy of 90.19% (70). The results are biased, most CNN algorithms are very prone to overfitting and memorizing the data, especially if the data-set is very small. Although it can be combined with wireless sensors to monitor heart rate and classify HRV from a distance, further research should be conducted with 50 people and larger datasets, in order to better verify the significance of developed algorithms for remote monitoring of HRV. The positive outcomes does hint that if researchers continue to improve existing CNN algorithms and the efficacy of analyzing data obtained through wireless sensors, remote monitoring of HRV can make a huge impact on the lives of others who are stressed due to work, suffering from cardiovascular diseases or are incapable of going to a clinician for routine checkups (90). The computational time complexity of convolutional layers is $0\left(n\right)=0\left(\overset{d}{\underset{l=1}{\sum }}n_{l-1}\cdot s_{l}^{2}\cdot n_{l}\cdot m_{l}^{2}\right)$, where *l* represents the index of the convolution layer, *d* represents the depth, *n*_*l*_ represents the number of filters in the *l*-th layer, *n*_*l*−1_ describes the number of input channels, *s*_*l*_ indicates the spatial size of the filter and *m*_*l*_ represents the spatial size of the output feature map (91). A typical 1D convolutional layer has a computational complexity of 0(*n*·*k*·*d*), further demonstrating the high computational resources and time required for a basic CNN architecture (92). Outside of HRV, there are numerous research conducted to reduce the computational cost of CNN, which typically compromises the output and classification accuracy (93). Inouchi et al. (93) developed a functionally-predefined kernel which significantly reduced the number of training parameters without compromising the accuracy. Further contribution toward similar methods catered toward HRV research can create a significant change within the healthcare system, such as reducing the number tedious hours needed from healthcare professionals and improving patient outcomes while decreasing healthcare costs.

## 7. Conclusion

This article which presented various summaries and reviews of the different applications associated with HRV research emphasized that reduced HRV is associated with increased morbidity and stress. Lower HRV is associated with increased SNS activity, which increases HR and blood pressure, presenting an immediate indication of the threat perceived by the ANS, which reacts to maintain normal function of the body and keep the body in a state of homeostasis. HRV in motion is less efficient in comparison to many other research studies such as stress and myocardial infarction. Numerous studies have indicated the lack of accuracy associated with exercise and drowsiness detection, this aspect of HRV research requires more attention and should be improved, in order to prevent injuries which may occur from performance fatigue near a sports competition or accidents associated with drowsy driving. HRV research will continue to expand due to its relevance in science, health and wellness of the heart. ML algorithms, AI (artificial intelligence) and frequency domain analysis of HRV can cause a huge impact in people's lives in a short period, if it is accurate, thus researchers go with the flow and improve these processing methods to improve lives/health of patients, prevent possible road accidents and enhance the quality of life.

### 7.1. Future Direction

HRV is a prominent topic concerning the activity of the heart and the ANS, although research has been steadily increasing, data analysis of HRV in motion is far from where it should be especially concerning drowsiness. Vicente et al. (23) and Georgiou et al. (21) have explained that HRV is hard to detect in motion, whether it involves exercise or drowsy driving, accuracy of HRV detection declines due to motion. Detection method in motion is a concern and should be a priority for improvement with regards to future research involving HRV. Machine learning algorithms, frequency domain analysis have been effective for stress analysis and remote monitoring of cardiovascular diseases through HRV analysis. Expansion in these domains of data analysis could provide effective/efficient results that produce an accurate representation of a person's HRV, which is easy to compute and can analyse a lot of data at once, making the detection process a lot smoother and quicker. Machine learning can be utilized to improve prognosis, since it can better assess medical records through logical algorithms in comparison currents scoring tools, which utilize a generalized thought process. CNN is a great algorithm that can effectively predict pathologies from X-ray images, at a faster rate than radiologists. Recent development also suggests that machine learning algorithms can create an immense impact toward public health, antedating infectious diseases and increasing the chances of preventing a chronic outcome.

## Author Contributions

All authors listed have made a substantial, direct and intellectual contribution to the work, and approved it for publication.

## Conflict of Interest

The authors declare that the research was conducted in the absence of any commercial or financial relationships that could be construed as a potential conflict of interest.
